# Quantitative detection of BK virus in kidney transplant recipients: a prospective validation study

**DOI:** 10.1590/1678-4685-JBN-3776

**Published:** 2018-04-19

**Authors:** Gabriel Godinho Pinto, José Antonio Tesser Poloni, Diego D'Avila Paskulin, Fabio Spuldaro, Fernanda de Paris, Afonso Luís Barth, Roberto Ceratti Manfro, Elizete Keitel, Alessandro C. Pasqualotto

**Affiliations:** 1 Universidade Federal de Ciências da Saúde de Porto Alegre, Porto Alegre, Brasil.; 2 Santa Casa de Misericórdia de Porto Alegre, Porto Alegre, Brasil.; 3 Hospital de Clínicas de Porto Alegre, Porto Alegre, Brasil.

**Keywords:** Kidney Transplantation, Viremia, Polymerase Chain Reaction, Polyomavirus, Transplante Renal, Viremia, Reação em Cadeia da Polimerase, Poliomavírus

## Abstract

**Introduction:**

BK virus (BKV) infection in renal transplant patients may cause kidney allograft dysfunction and graft loss. Accurate determination of BKV viral load is critical to prevent BKV-associated nephropathy (BKVAN) but the cut-off that best predicts BKVAN remains controversial.

**Objective:**

To evaluate the performance of a commercial and an in-house qPCR test for quantitative detection of BK virus in kidney transplant recipients.

**Methods:**

This was a prospective study with kidney transplant recipients from two large university hospitals in Brazil. Patients were screened for BKV infection every 3 months in the first year post-transplant with a commercial and an in-house real time polymerase chain reaction (qPCR) test. BKVAN was confirmed based on histopathology. The area under the curve for plasma qPCR was determined from receiver operating characteristic analysis.

**Results:**

A total of 200 patients were enrolled. Fifty-eight percent were male, 19.5% had diabetes mellitus, and 82% had the kidney transplanted from a deceased donor. BKV viremia was detected in 32.5% and BKVAN was diagnosed in 8 patients (4%). BKVAN was associated with viremia of 4.1 log copies/mL, using a commercial kit. The cut-off for the in-house assay was 6.1 log copies/mL. The linearity between the commercial kit and the in-house assay was R^2^=0.83.

**Conclusion:**

Our study shows that marked variability occurs in BKV viral load when different qPCR methodologies are used. The in-house qPCR assay proved clinically useful, a cheaper option in comparison to commercial qPCR kits. There is an urgent need to make BKV standards available to the international community.

## Resumo

**Introdução:**

A infecção pelo vírus BK (BKV) em pacientes de transplante renal pode levar a disfunção do aloenxerto renal e perda do enxerto. A determinação precisa da carga viral do BKV é fundamental para prevenir a nefropatia associada ao BKV (BKVAN), mas o ponto de corte de melhor valor preditivo para BKVAN ainda é foco de debates.

**Objetivo:**

Avaliar o desempenho de um teste de qPCR comercial e outro desenvolvido internamente para detecção quantitativa de vírus BK em receptores de transplante renal.

**Métodos:**

O presente estudo prospectivo incluiu receptores de transplante renal de dois grandes hospitais universitários no Brasil. Os pacientes foram testados para infecção por BKV a cada três meses no primeiro ano pós-transplante com um teste comercial de reação em cadeia de polimerase quantitativa em tempo real (qPCR) e outro desenvolvido internamente. A presença de BKVAN foi confirmada com base na histopatologia. A área sob a curva para o qPCR plasmático foi determinada a partir da análise da característica de operação do receptor.

**Resultados:**

Um total de 200 pacientes foram incluídos. Cinquenta e oito por cento eram do sexo masculino, 19,5% tinham diabetes mellitus e 82% tiveram seus rins transplantados de doadores falecidos. Viremia de BKV foi detectada em 32,5% dos pacientes e oito (4%) foram diagnosticados com BKVAN. BKVAN foi associada a viremia de 4,1 log cópias/mL usando o kit comercial. O corte para o ensaio interno foi de 6,1 log cópias/mL. A linearidade entre o kit comercial e o ensaio interno foi R^2^ = 0,83.

**Conclusão:**

Nosso estudo demonstrou uma acentuada variabilidade na carga viral de BKV quando diferentes metodologias de qPCR foram utilizadas. O ensaio interno de qPCR mostrou-se clinicamente útil, além de ser uma opção menos onerosa em relação aos kits comerciais de qPCR. Há uma necessidade urgente de se definir padrões de BKV para a comunidade internacional.

## Introduction

BK virus (BKV) is an important infection agent in renal transplant recipients, which has the potential to cause severe graft dysfunction and eventually graft loss .^1^ The prevalence of BKV-associated nephropathy (BKVAN) in renal transplant patients ranges between 1-10% in the first year after transplantation, and graft loss may occur in up to 80% of these individuals.^2^^-^ 4 BKV is usually acquired early in life via aerosols mostly resulting in asymptomatic infection. It is estimated that 80-90% of the adult population present antibodies against BKV.^3^^,^ 7 ^,^ 8

Since no effective antiviral therapy is available to treat BKV infection, the best strategy relies on BKVAN prevention. This may be achieved by frequent monitoring of BKV DNA load in urine and/or plasma samples, followed by a reduction of immunosuppressive therapy whenever significant viral replication is detected.^9^ International societies have recommended 4 log of BKV DNA in the plasma as the cut-off value that best predicts BKVAN. However, commercial tests based on quantitative real time polymerase chain reaction (qPCR) may be expensive for routine use in clinical practice and limited data is available on the performance of in-house qPCR BKV tests. Therefore, it is critical for institutions to conduct clinical validation studies to certify that their methods are useful to accurately guide clinical decisions.^10^^-^ 17

The purpose of this study was to establish a clinically significant cut-off value for BKV viremia to predict BKVAN in a cohort of renal transplant recipients. We also report the performance of an in-house qPCR for quantification of BKV viral load and the performance of this test in comparison to a commercially available qPCR kit.

## Material And Methods

### Samples

Between April 2012 and May 2013, 200 patients that received a kidney transplant in two large Brazilian university hospitals (Santa Casa de Misericórdia de Porto Alegre and Hospital de Clínicas de Porto Alegre) were enrolled in a prospective study. Plasma samples were obtained at months 3, 6, and 9 following kidney transplantation for the determination of BKV viral load. DNA was extracted from 140 µL of plasma using the QIAamp RNA Mini Kit (QIAGEN, USA). In all reactions, β-globin was added as an internal positive control.

### DNA amplification with a commercial QPCR kit

BKV DNA amplification was performed by qPCR using a commercial kit (BKV Q-PCR Alert Ampliprobe, ELITechGroup Nanogen, Buttigliera Alta, Italy) in a 7500 thermal cycler qPCR System (Applied Biosystems), as previously described.^18^

### DNA amplification with an in-house BKV QPCR test

We designed a qPCR assay based on TaqMan chemistry in a highly conserved region of the BKV genome targeting the VP1 gene (Gene ID: 1489515, Genomic Sequence NC_001538.1) with Primers 5’-AGTGTTGAGAATCTGCTGTTGCTT-3’ and5’-GGGATGAAGATTTATTTTGCCATGAAGAT-3’; probe FAM-CATCACTGGCAAACAT-NFQ). Primers and probes for the human acidic ribosomal protein (HuPO) were purchased from Applied Biosystems (ABI) (primers 5’-GACAATGGCAGCATCTACAAC-3’ and 5’-GTTGCCAGTGTCTGTCTGC-3’; probe FAM-ATTGCGGACACCCTCC-NFQ) and were used as an internal control. Briefly, the in-house qPCR assay consisted of 1 µL 20X TaqMan assay, 10 µL of 2X TaqMan® Gene Expression Master Mix, 4 µL of DNA and 5 µL of RNase-free water. PCR amplification was performed on an ABI 7500 Thermocycler as follows: 95ºC for 10 min, and 40 cycles of 95ºC for 15 sec and 60ºC for 1 min.

In order to accurately build a calibration curve, we designed a synthetic DNA sequence of 351 bp based on the BK polyomavirus GenBank strain JQ713822.1 sequence. The synthetic DNA was eluted, quantified, and serially diluted for the calibration curves that were built as a panel of nine vials with concentrations ranging from 12.9 to 12.9 × 109 copies/mL. The detection limit of the assay was determined as 12.9 genomic copies/mL.

### Clinical Data

Patients’ records were reviewed to obtain clinical data and demographic information. Variables of interest included underlying kidney diseases, HLA mismatches, renal biopsy results, and changes in immunosuppressive regimens. Renal biopsies were performed by clinical indication. The glomerular filtration rate (GFR) was estimated using the CKP-EPI equation.^19^ The study was approved by the Institutional Review Board (protocol numbers 3531/11, 12-154 and 915/12), and followed the guidelines and regulatory standards for research involving human subjects of the Brazilian National Health Council (Resolution CNS/196).

### Statistical Analysis

Descriptive statistics were used to summarize the data. The chi-square and Fisher exact tests were used for the evaluation of categorical variables. Data normality was checked by Kolmogorov-Smirnov test. Normally distributed scalar variables were analyzed using ANOVA or Student t-test as appropriate. Non-normally distributed scalar variables were analyzed as non-parametric using the Mann-Whitney test. The performance of qPCR tests was evaluated by receiver operating characteristic (ROC) curves, using kidney biopsy as the gold standard to diagnose BKVAN. Linear plots were built to test the linearity between the commercial and the in-house BKV qPCR tests. For all comparisons, statistical significance was determined at a p value of <0.05. Predictors of BKVAN development were determined in a Cox regression model. All variables with clinical relevance and p values of ≤0.05 at univariate analysis were included in the Cox regression model. Statistical analyses were performed using SPSS 20.0.

## Results

Table 1 summarizes the main characteristics of the patients enrolled in the study. Kidney transplant patients who developed BKV infection along the study period were similar in several aspects to those who did not. Panel reactive antibodies (PRA) differed between groups. BKV-positive group had lower percentages of patients with PRA <10% and between 10-49%. Distribution of HLA mismatches did not differ between groups. Ninety-nine patients underwent a renal biopsy and eight (4.0%) developed BKVAN. Graft loss occurred in seven patients (3.5%) but BKVAN was considered the cause for graft loss in only one patient (14.3%; overall incidence 0.5%). Seven patients died during the study (3.5%).

**Table 1:** Demographic and clinical characteristics of the population studied. Patient's BKV status refers to the presence of any positive molecular test for plasma BKV

Variable	All patients (n=200)	BKV-negative patients (n=75)	BKV-positive patients (n=125)	p-value
Recipient age, mean (sd)	46.3 (13.2)	47.4 (12.9)	45.7 (13.4)	0.659
Donor age, mean (sd)	44.5 (16.3)	43.5 (17.3)	45.2 (15.8)	0.409
Male gender, recipient (%)	58.0	54.7	60	0.459
Male gender, donor (%)	52.0	51.4	52.8	0.843
Deceased donor (%)	82.0	82.4	81.6	0.883
Underlying disease (%)				
Diabetes mellitus	19.5	26.7	15.2	0.048
ADPKD	13.5	9.3	16	0.182
SAH	12.0	13.3	11.2	0.653
Glomerulonephritis	10.0	12	8.8	0.465
Reflux nephropathy	6.0	6.7	5.6	0.758
Obstructive uropathy	2.5	2.7	2.4	0.907
FSG	2.5	1.3	3.2	0.413
SLE	1.5	0	2.4	0.176
Unknown	32.5	28	35.2	0.293
Indução (%)				
ATG	32.5	41.3	27.2	0.039
Baxiliximab	56.5	49.3	60.8	0.113
Others	0.5	0	0.8	0.437
None	10.5	9.3	11.2	0.677
PRA (%)				
Class I				
<10%	64.0	55.4	69	0.033
≥10% to <50%	22.0	31.1	16.7	0.037
≥50%	14.0	13.5	14.3	0.641
Class II				
<10%	62.0	53.3	67.2	0.050
≥10% - <50%	31.0	42.7	24	0.006
≥50%	7.0	4	8.8	0.198
CMV Status (%)				
D- / R-	1.6	0	2.5	0.185
D- / R+	17.2	17.4	17.5	0.995
D+ / R-	4.2	4.3	4.2	0.943
D+ / R+	76.7	78.3	75.8	0.634
+ve antigenemia	25.0	18.7	29.4	0.111
HLA Mismatch, mean (sd)	4.2 (1)	4.2 (1.3)	4.6 (1.3)	0.758
0 (%)	0.5	0	0.8	0.437
1 - 3 (%)	22	29.3	17.6	0.169
4 - 6 (%)	77.5	70.7	81.6	0.730
DSA (%)	11.6	22.7	4.8	<0.001
Acute rejection (%)	12.5	19.5	29.3	0.269

Legend: ADPKD, Autossomal dominant polycystic kidney disease; ATG, Anti-Thymocyne globulin; BKV, BK virus; CMV, Cytomegalovirus; D, Donor; DSA, Donor-specific antibody; FSG, Focal segmental glomerulosclerosis; HLA, Human leukocyte antigen; PRA, Panel reactive antibody; R, Recipient; sd, standard deviation; SAH, Systemic Arterial Hypertension; SLE, Systemic lupus erythematosus.

### Performance of the commercial and the in-house QPCR test

BKV viremia was detected in 32.5% (66/200) of patients using the commercial qPCR kit. BKV viremia was detected in months 3, 6 and 9 following transplantation in 16.5% (n = 33), 19.4% (n = 34), and 12.3% (n = 18) of patients, respectively. Plasma BKV viral load was higher in BKVAN when compared to non-BKVAN patients (*p* <0.05).

**Figure 1:**
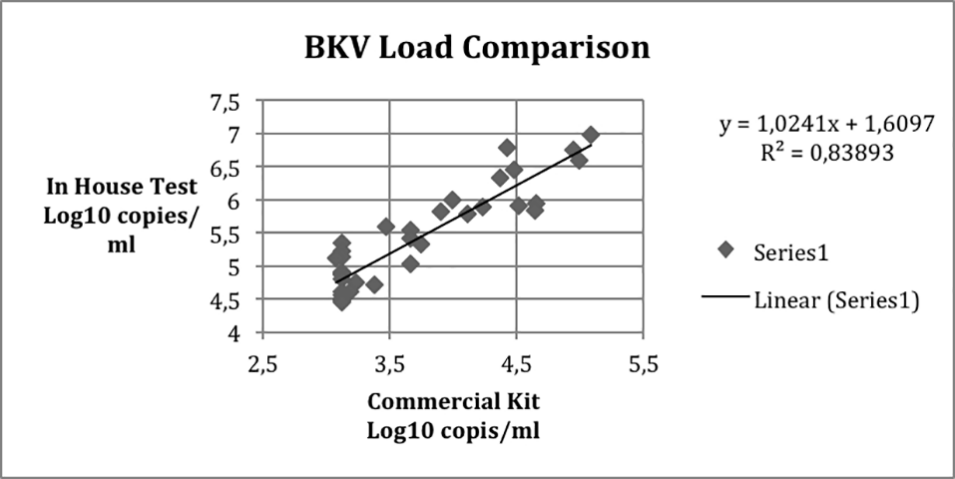
Linear relationship between qPCR in house test and commercial kit test.

Table 2 shows the cut-off values of qPCR for the prediction of BKVAN, both for the commercial qPCR test and the in-house PCR test. There was a linear relationship between qPCR tests (R^2^=0.8389) (Figure 1).

**Table 2:** Performance of BKV viral load for the prediction of BKV-associated nephropathy, using a commercial and an in-house QPCR test

	Sensitivity % (95% CI)	Specificity % (95% CI)	PPV % (CI 95%)	NPV % (CI 95%)
Plasma viral load (copies/ml)				
≥3.8 log (commercial PCR kit)	88 (47-98)	96 (90-99)	64 (31-89)	99 (94-100)
≥4.1 log (commercial PCR kit)	88 (47-98)	98 (93-100)	77 (40-97)	99 (94-100)
≥6.1 log (in-house method)	87 (81-93)	100 (94-100)	73 (39-94)	100 (91-100)

Legend: CI, confidence interval; NPV, negative predictive value; PPV, positive predictive value; qPCR, quantitative real-time polymerase chain reaction.

### Predictors of BKVAN

Table 3 shows the results of the multivariate analysis for risk factors for BKVAN using the commercial qPCR kit. BKV viremia was independently associated with BKVAN (*p* = 0.018), with the best cut-off value determined at 3.85 log (7169 copies/mL).

**Table 3:** Results of Cox regression model for the prediction of BKVAN using a commercial QPCR test

	p-value	Exp(B)	CI 95,0% for Exp(B)	
			lower	upper
Acute rejection	0.487	2.846	0.149	54.214
Diabetes mellitus	0.324	2.658	0.38	18.578
Viremia at 3.85 log	0.018	35.903	1.85	696.868
GFR at month 6	0.512	1.521	0.434	5.328

Legend: CI, Confidence interval; GRF, Glomerular rate filtration; qPCR, quantitative real time polymerase chain reaction.

## Discussion

This study defined clinically significant cut-off values for qPCR for the prediction of BKVAN, using two molecular tests: a commercially-available qPCR kit and an in-house qPCR test. At nine months post-transplantation, 32.5% of patients were found to have BKV viremia, but only 8 (4%) developed BKVAN. Previous studies performed in Brazil showed higher frequencies of BKVAN in kidney transplant recipient,^20^^,^ 21 which may be related to differences in screening strategy (e.g., urinary decoy cells to trigger additional urine/plasma sampling for qPCR), in addition to regular biopsies, and ischemia times. The most relevant implication of BKV infection in renal transplant recipients relies on its ability to lead to graft fibrosis, which can be followed by renal dysfunction and eventually lead to graft loss.^13^ Therefore, in order to correctly interpret BKV viral loads, institutions should validate their own methodologies to determine the optimum cut-off values instead of using the recommended ‘universal’ cut-off of 4 log copies/mL as being clinically significant.^3^ For accurate BKV viral load interpretation, clinicians should know which PCR test was used and how it performs. Several studies have analyzed the clinical impact of BKV viremia using diverse methodologies. Therefore, cut-offs generated with different qPCR assays cannot be compared against each other due to marked methodological variability.^10^^-^ 17 A variety of factors contribute to this diversity, including different protocols for DNA extraction, variations in primers and probes design, viral targets, PCR conditions, sample type, and the use of different calibration curves.^17^^,^ 22 ^,^ 23

In this study we demonstrated that BKV viremia can predict the occurrence of BKVAN, and that different cut-offs need to be applied to different qPCR assays (≥3.8 log and ≥6.1 log copies/mL respectively for the commercial and in-house kits). Also, our study demonstrated the relationship between PRA and BKV infection considering patients with PRA <50% of class I and II, although PRA ≥50 presented no association with BKV (Table 1). These data contrast with previous studies, which considered that PRA ≥10% was not associated with BKV infection.^24^ In our cohort 11.6% of patients had DSA, however 69% of these patients did not evolved to BKV infection (*P* ≤ 0.001). Our in-house qPCR test has several strengths: (i) it was based on a highly conserved region of the BKV genome targeting the viral structural protein VP1 gene that is highly conserved midst BKV strains;^25^ and (ii) the quantitative process was based on the use of a synthetic DNA sequence as a calibration curve, therefore not requiring the use of commercially available quantified BKV DNA controls. Results obtained with the in-house qPCR test showed linearity with the commercial kit (ELITechGroup Nanogen, Italy), although cut-off values differed by ~2 log copies/mL. Probably the main advantage of the in-house qPCR relies on its reduced cost, in comparison to the commercial test. For instance, the costs related to run a single sample is USD 35 and USD 121, respectively for the in-house qPCR test and the commercial kit. If three samples were included in a run, reducing the expenses with positive controls, costs per sample would be USD 20 (in-house qPCR) and USD 55 (commercial test).

Some limitations of this study must be recognized. The number of patients with BKVAN was limited even though the frequency of BKVAN in this study parallels with what is found in the literature.^2^^,^ 3 Also, we only measured BKV viral loads at months 3, 6 and 9 after transplantation and perhaps a longer follow-up could demonstrate a higher incidence of BKVAN, even though the peak incidence of BKVAN occurs within the time frame of our observation.^26^^,^ 27

In conclusion, in this prospective multicenter study we validated clinically two qPCR assays for BKV quantification, a commercially available kit and an in-house test. Based on the results, clinicians may better manage patients infected with BKV, modifying immunosuppressive therapies in a timely manner. The low frequency of BKVAN observed in our study (4%) is probably related to proper disease awareness, as well as BKV DNA monitoring.

## References

[1] van Aalderen MC, Heutinck KM, Huisman C, ten Berge IJ (2012). BK virus infection in transplant recipientes: clinical
manifestations, treatment options and the immune response. Neth J Med.

[2] Cannon RM, Ouseph R, Jones CM, Hughes MG, Eng M, Marvin MR (2011). BK viral disease in renal transplantation. Curr Opin Organ Transplant.

[3] Hirsch HH, Brennan DC, Drachenberg CB, Ginevri F, Gordon J, Limaye AP (2005). Polyomavirus-associated nephropathy in renal transplantation:
Interdisciplinary analyses and recommendations. Transplantation.

[4] Egli A, Binggeli S, Bodaghi S, Dumoulin A, Funk GA, Khanna N (2007). Cytomegalovirus and polyomavirus BK
posttransplant. Nephrol Dial Transplant.

[5] Siguier M, Sellier P, Bergmann JF (2012). BK-virus infections: a literature review. Med Mal Infect.

[6] Shah KV (2000). Human polyomavirus BKV and renal disease. Nephrol Dial Transplant.

[7] Brennan DC, Agha I, Bohl DL, Schnitzler MA, Hardinger KL, Lockwood M (2005). Incidence of BK with tacrolimus versus cyclosporine and impact of
preemptive immunosuppression reduction. Am J Transplant.

[8] Knowles WA, Pipkin P, Andrews N, Vyse A, Minor P, Brown DW (2003). Population-based study of antibody to the human polyomaviruses
BKV and JCV and the simian polyomavirus SV40. J Med Virol.

[9] Kuypers DR (2012). Management of polyomavirus-associated nephropathy in renal
transplant recipients. Nat Rev Nephrol.

[10] Bechert CJ, Schnadig VJ, Payne DA, Dong J (2010). Monitoring of BK viral load in renal allograft recipients by
real-time PCR assays. Am J Clin Pathol.

[11] Hassan S, Mittal C, Amer S, Patel A, Delbusto R, Samuel L (2014). Currently recommended BK virus (BKV) plasma viral load cutoff of
≥ 4 log10/mL underestimates the diagnosis of BKV-associated nephropathy: a
single transplant center experience. Transpl Infect Dis.

[12] Hirsch HH, Drachenberg CB, Steiger J, Ramos E (2006). Polyomavirus-associated nephropathy in renal transplantation:
critical issues of screening and management. Adv Exp Med Biol.

[13] Hirsch HH, Knowles W, Dickenmann M, Passweg J, Klimkait T, Mihatsch MJ (2002). Prospective study of polyomavirus type BK replication and
nephropathy in renal-transplant recipients. New Eng J Med.

[14] Kudose S, Dong J (2014). Clinical validation study of quantitative real-time PCR assay for
detection and monitoring of BK virus nephropathy. Ann Clin Lab Sci.

[15] Mitui M, Leos NK, Lacey D, Doern C, Rogers BB, Park JY (2013). Development and validation of a quantitative real time PCR assay
for BK virus. Mol Cell Probes.

[16] Pollara CP, Corbellini S, Chiappini S, Sandrini S, De Tomasi D, Bonfanti C (2011). Quantitative viral load measurement for BKV infection in renal
transplant recipients as a predictive tool for BKVAN. New Microbiol.

[17] Randhawa P, Kant J, Shapiro R, Tan H, Basu A, Luo C (2011). Impact of genomic sequence variability on quantitative PCR assays
for diagnosis of polyomavirus BK infection. J Clin Microbiol.

[18] Pinto GG, Poloni JA, Carneiro LC, Baethgen LF, Barth AL, Pasqualotto AC (2013). Evaluation of different urine protocols and DNA extraction
methods for quantitative detection of BK viruria in kidney transplant
patients. J Virol Methods.

[19] Arlet JB, Ribeil JA, Chatellier G, Eladari D, De Seigneux S, Souberbielle JC (2012). Determination of the best method to estimate glomerular
filtration rate from serum creatinine in adult patients with sickle cell
disease: a prospective observational cohort study. BMC Nephrol.

[20] Zalona AC, Varella RB, Takiya CM, Goncalves RT, Zalis MG, Santoro-Lopes G (2013). A qualitative seminested PCR assay as an alternative to urine
cytology for BK polyomavirus screening after renal
transplantation. Intervirology.

[21] Maia TM, Silva SF, Silva SL, Holanda MC, Nascimento JM, Ferreira MV (2011). Polyomavirus-infected decoy cells in cytocentrifuged urine
cytology specimens from renal transplant recipients. Acta Cytol.

[22] Hayden RT, Yan X, Wick MT, Rodriguez AB, Xiong X, Ginocchio CC, College of American Pathologists Microbiology Resource
Committee (2012). Factors contributing to variability of quantitative viral PCR
results in proficiency testing samples: a multivariate
analysis. J Clin Microbiol.

[23] Hoffman NG, Cook L, Atienza EE, Limaye AP, Jerome KR (2008). Marked variability of BK virus load measurement using
quantitative real-time PCR among commonly used assays. J Clin Microbiol.

[24] Awadalla Y, Randhawa P, Ruppert K, Zeevi A, and Duquesnoy RJ (2004). HLA mismatching increases the risk of BK virus nephropathy in
renal transplant recipients. Am J Transplant.

[25] Krumbholz A, Bininda-Emonds OR, Wutzler P, Zell R (2008). Evolution of four BK virus subtypes. Infect Genet Evol.

[26] Girmanova E, Brabcova I, Bandur S, Hribova P, Skibova J, Viklicky O (2011). A prospective longitudinal study of BK virus infection in 120
Czech renal transplant recipients. J Med Virol.

[27] Huang G, Chen LZ, Qiu J, Wang CX, Fei JG, Deng SX (2010). Prospective study of polyomavirus BK replication and nephropathy
in renal transplant recipients in China: a single-center analysis of
incidence, reduction in immunosuppression and clinical
course. Clin Transplant.

